# Cellular inhibitor of apoptosis 2 (cIAP2) restricts neuroinflammation during experimental autoimmune encephalomyelitis

**DOI:** 10.1186/s12974-022-02527-6

**Published:** 2022-06-19

**Authors:** Debolina D. Biswas, Rebecca K. Martin, LaShardai N. Brown, Karli Mockenhaupt, Angela S. Gupta, Michael J. Surace, Anuj Tharakan, Jessie W. Yester, Reetika Bhardwaj, Daniel H. Conrad, Tomasz Kordula

**Affiliations:** 1 Department of Biochemistry and Molecular Biology, School of Medicine and the Massey Cancer Center, Virginia Commonwealth University, Richmond, VA 23298 USA; 2 Department of Microbiology and Immunology, School of Medicine and the Massey Cancer Center, Virginia Commonwealth University, Richmond, VA 23298 USA

**Keywords:** EAE, Neuroinflammation, cIAP2, Microglia, Caspase-8

## Abstract

**Background:**

Immune activation, neuroinflammation, and cell death are the hallmarks of multiple sclerosis (MS), which is an autoimmune demyelinating disease of the central nervous system (CNS). It is well-documented that the cellular inhibitor of apoptosis 2 (cIAP2) is induced by inflammatory stimuli and regulates adaptive and innate immune responses, cell death, and the production of inflammatory mediators. However, the impact of cIAP2 on neuroinflammation associated with MS and disease severity remains unknown.

**Methods:**

We used experimental autoimmune encephalomyelitis (EAE), a widely used mouse model of MS, to assess the effect of cIAP2 deletion on disease outcomes. We performed a detailed analysis on the histological, cellular, and molecular levels. We generated and examined bone-marrow chimeras to identify the cIAP2-deficient cells that are critical to the disease outcomes.

**Results:**

cIAP2^−/−^ mice exhibited increased EAE severity, increased CD4^+^ T cell infiltration, enhanced proinflammatory cytokine/chemokine expression, and augmented demyelination. This phenotype was driven by cIAP2-deficient non-hematopoietic cells. cIAP2 protected oligodendrocytes from cell death during EAE by limiting proliferation and activation of brain microglia. This protective role was likely exerted by cIAP2-mediated inhibition of the non-canonical NLRP3/caspase-8-dependent myeloid cell activation during EAE.

**Conclusions:**

Our findings suggest that cIAP2 is needed to modulate neuroinflammation, cell death, and survival during EAE. Significantly, our data demonstrate the critical role of cIAP2 in limiting the activation of microglia during EAE, which could be explored for developing MS therapeutics in the future.

**Supplementary Information:**

The online version contains supplementary material available at 10.1186/s12974-022-02527-6.

## Background

Neuroinflammation develops in the central nervous system (CNS) in response to injury, infection, autoimmune response, or metabolic stress to prevent exacerbated damage and to allow for the return to homeostasis [1–4]. Initially, this response relies on the rapid and non-specific activation of microglia that are the resident innate immune cells of the brain [5]. Subsequently, B cells and T cells of the adaptive immune system are recruited providing a highly specific, but much slower response [4, 6]. Recruitment of these cells is tightly controlled especially at the onset of neuroinflammation because of the presence of a blood–brain barrier that separates the CNS from the rest of the body. In addition, non-immune cells, including astrocytes, endothelial cells, and oligodendrocytes modulate neuroinflammation [4, 7–10]. While neuroinflammation is critical for tissue repair, unresolved chronic neuroinflammation, with persistent activation of microglia and increased expression of proinflammatory cytokines and chemokines, can lead to neurodegeneration as manifested in Alzheimer’s, Parkinson’s, and Huntington’s diseases, amyotrophic lateral sclerosis, and multiple sclerosis (MS) [11–13]. MS is an inflammatory autoimmune demyelinating disease of the CNS characterized by muscle weakness, impaired coordination, and sensory loss. While the primary cause of the disease remains elusive, demyelination, primarily mediated by autoreactive T cells recognizing myelin, and persistent inflammation cause damage to the CNS. Lesions that develop in MS patients are characterized by immune cell infiltration, loss of oligodendrocytes and myelin-associated glycoproteins, activation of glial cells, and axon degeneration [1, 2].

Inhibitors of apoptosis (IAPs) belong to a family of highly conserved proteins, containing the baculoviral IAP repeat (BIR), found in many organisms, including yeast, insects, fish, and mammals [14, 15]. Although IAPs are anti-apoptotic and regulate cell death [16], they also act as critical cell signaling regulators [17]. Mammalian cellular IAP1 (cIAP1), cIAP2, X-linked IAP (XIAP), melanoma IAP, and IAP-like protein 2 contain the RING domain and function as E3-ubiquitin ligases catalyzing both polyubiquitylation and monoubiquitylation [18–21]. Three unique BIR domains and a single ubiquitin-binding domain are present in cIAP1, cIAP2, and XIAP [14]. In addition, cIAP1 and cIAP2 contain caspase activation and recruitment domain [22]. cIAP1, cIAP2, and XIAP are potent regulators of both the innate and adaptive immune responses regulating signaling pathways activating transcription factor nuclear factor kB (NF-κB) and mitogen-activated protein kinase (MAPK) [14]. cIAP1 and cIAP2 function downstream of the tumor necrosis factor receptor 1 (TNFR), toll-like receptors (TLR), nucleotide-binding oligomerization domain (NOD) receptors, and several cytokine receptors [23, 24]. Both cIAP1 and cIAP2 bind and function with an adaptor, Tumor Necrosis Factor Receptor (TNFR)-Associated Factor 2 (TRAF2), which is required for their stabilization, recruitment to the ligand-bound receptors, and localization within the cell [25, 26]. cIAP1/2 mediate ubiquitylation of various signaling components, including receptor-interacting kinase 1 (RIPK1), apoptotic caspases, an inhibitor of NF-κB kinase gamma (IKKγ) and IKKε, NF-kB-inducing kinase (NIK), and transcription factors, such as c-Rel, interferon regulatory factor 1 (IRF1), and IRF5 [27–32]. Importantly, cIAP1/2 not only regulate apoptosis and inflammatory signaling, but also limit activation of RIPK1/3-mediated necroptosis depending on the presence of the other regulatory proteins, such as caspase-8, and cellular FLICE-like-inhibitory protein [33–35]. Deletion of both cIAP1 and cIAP2 in mice induces inflammation and leads to death that can be partially rescued by deletion of caspase-8 [35], suggesting that a major function of cIAP1/2 is suppression of caspase-8-dependent cell death. In contrast to constitutively expressed cIAP1, cIAP2 expression is induced in response to many inflammatory stimuli [36–38], suggesting its unique functions. cIAP2^−/−^ mice are protected from lipopolysaccharide (LPS)-induced death due to increased macrophage apoptosis and diminished inflammatory responses [39]. However, cIAP2^−/−^ mice are more susceptible to *Listeria monocytogenes* infection because of increased macrophage necroptosis [34]. cIAP2 also orchestrates intestinal homeostasis with cIAP2^−/−^ mice being more susceptible to acute and chronic colitis due to increased cell death and impaired activation of regenerative interleukin 18 [40]. Furthermore, cIAP2, but not cIAP1, mediates polyubiquitylation of IL-1-induced IRF1 that stimulates expression of chemokines needed to recruit adaptive immune cells to the site of inflammation [31].

Immune activation, neuroinflammation, and cell death are the hallmarks of MS. Although cIAP2 is one of the known regulators of these processes, its impact on neuroinflammation and MS remains unknown. Furthermore, small-molecule antagonists, known as SMAC (second mitochondrial-derived activator of caspases) mimetics that promote IAP autoubiquitylation and the degradation [41, 42] are being developed for cancer therapy. This raises the question of their putative impact on neuroinflammation. In this work, we explored the role of cIAP2 on neuroinflammation associated with MS.

## Materials and methods

### Mice

cIAP2^−/−^ mice provided by Dr. Korneluk (University of Ottawa) were housed at Virginia Commonwealth University according to the guidelines of the Institutional Animal Care and Use Committee. The mouse protocols were approved by the Institutional Animal Care and Use Committee. Animals were housed in the animal facility, with a 12 h light/dark cycle, and provided water and standard laboratory chow ad libitum. Randomly chosen males and females were used for all experiments. All animals were included for data analysis unless they reached a set humane endpoint (20% weight loss) before the end of the experiment. The group sizes for each experiment are provided in figure legends. The disease progress was recorded for all experimental animals, while molecular analysis was performed in smaller animal groups.

### Induction and scoring of experimental autoimmune encephalomyelitis

EAE was induced in wildtype (WT) and cIAP2-deficient mice. Mice were immunized twice, on day 0 and day 7, subcutaneously with 250 μg MOG_35–55_ peptide (AnaSpec) emulsified in CFA containing 500 μg *Mycobacterium tuberculosis* H37Ra (Difco). On days 0 and 3, mice received intraperitoneally 200 ng Bordetella pertussis toxin (Enzo Life Sciences). Mice were weighed, and severity of the disease was scored daily for neurological signs using a five-point scale: 0, no symptoms; 1, limp tail; 2, limp tail with loss of righting; 3, paralysis of a single hind limb; 4, paralysis of both hind limbs; and 5, moribund state or death. Two–three independent experiments were performed (as indicated in figure legends), and cumulative data are presented. Tissues were collected at day 12 for molecular/cellular analysis.

### Bone-marrow reconstitution

Both WT and cIAP2^−/−^ mice were irradiated for 6 min 15 s at an intensity of 550 centi-Gy at an interval of 2 h. Bone marrow from naïve mice were harvested from the femur and tibia of the naïve wildtype and cIAP2^−/−^ mice. Five million bone-marrow cells were injected in the irradiated mice by tail vein. EAE was induced in these mice after 8 weeks of bone-marrow reconstitution.

### Isolation and flow cytometry analysis of CNS cells

**Fig. 1:**
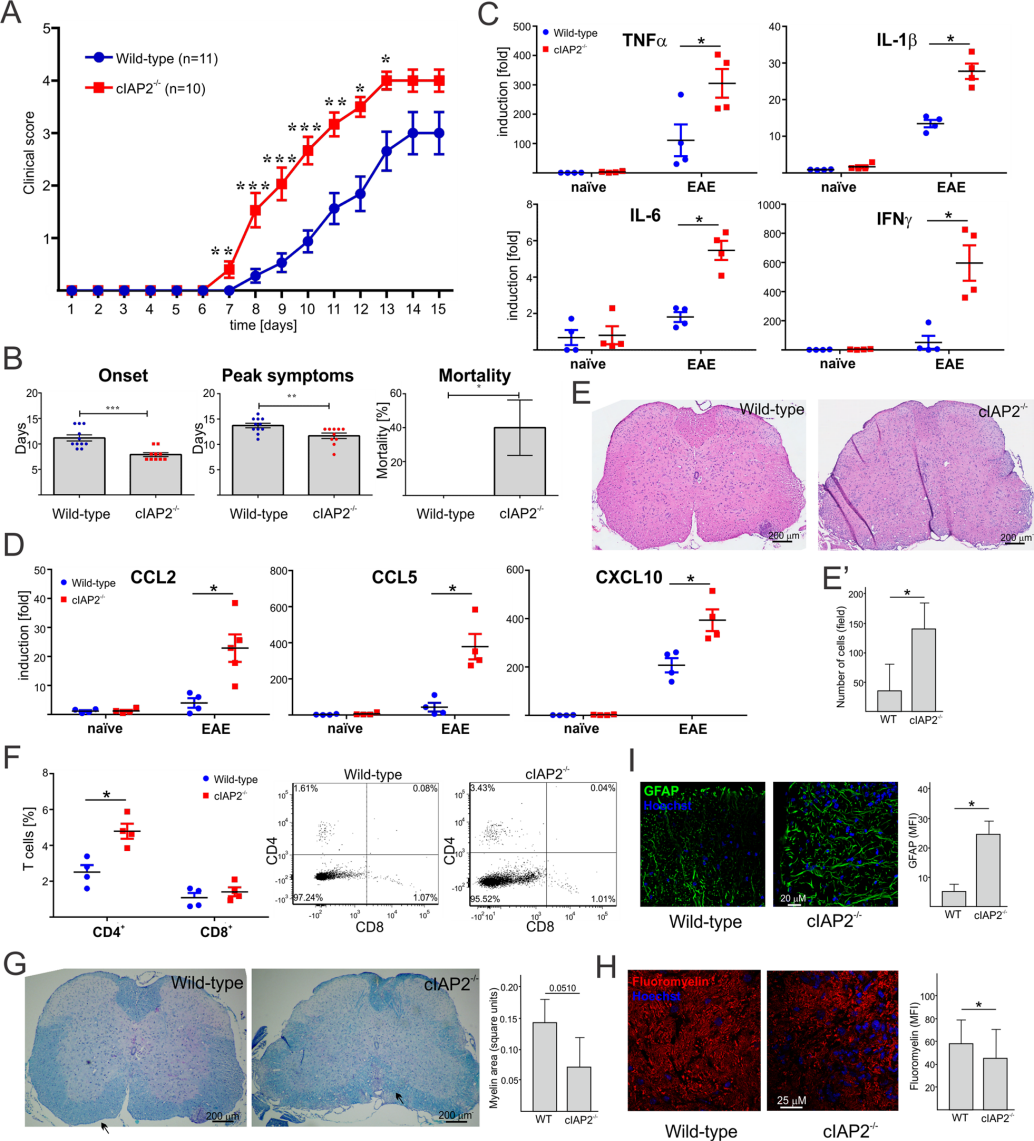
cIAP2 deficiency exacerbates EAE disease. **A** Mean clinical EAE scores in WT (n = 11) and cIAP2^−/−^ (*n* = 10) mice. Data are representative of two independent experiments. **B** Disease onset, peak symptoms, and percentage of mortality for WT (*n* = 11) and cIAP2^−/−^ (*n* = 10) mice. Inflammatory cytokine (**C**) and chemokine (**D**) mRNA expression in naïve (*n* = 4, 4) and EAE (*n* = 5, 5) lumbar spinal cords (day 12). **E** H&E staining of lumbar spinal cords (L2-L4) collected at day 12 of disease (representative images). **E’** Quantification of inflammatory cells infiltrating lumbar spinal cords (L2-L4) (6 areas each). **F** T cell infiltration. Flow cytometry gating of CD4^+^ and CD8^+^ T cells (initially gated as CD45^+^CD11^−^ lymphocytes) determined at day 12 of EAE in the WT (*n* = 4) and cIAP2^−/−^ (*n* = 4) brains. **G** Luxol Fast Blue (Quantification; 5 areas each), Fluoromyelin (**H**) (Quantification; 50 areas each), and GFAP **I** (Quantification; 15 areas each), staining of lumbar spinal cords (L2-L4) collected at day 12 of disease (representative images). **A–D**, **F** Mean ± SEM, *p < 0.05, **p < 0.01, ***p < 0.001, *T*-test

Isolation of cells and their analysis were previously described [43]. Briefly, brains of naïve (Additional file 3: Fig. S3B) or at day 12 of EAE (Figs. 1F and 4C) WT and cIAP2^−/−^ mice were homogenized using Wheaton Dounce glass tissue grinders. Cells were centrifuged at 1500 rpm for 5 min at 4 °C, resuspended in 10 ml of 30% Percoll (Amersham Bioscience), and centrifuged onto a 70% Percoll for 30 min at 2600 rpm. Cells collected at the 30–70% interface were strained through 70 µm filters and stained with fluorescence-conjugated monoclonal antibodies against CD45 (clone 30-F11), CD11b (clone M1/70), CD4 (clone GK 1.5), CD8 (clone 53–6.7), and Ly6C (clone HK1.4), F4/80 (clone BM8), and isotype control antibodies (Biolegend) were used to define specificity of the staining. Fluorescence data were collected on a BD LSRFortessa-X20 and acquired using BD FACS Diva 8 software and analyzed.

### Isolation of microglia from EAE brains

The microglia were isolated from the brains of EAE-induced mice after 12 days of immunization as described previously [44]. In brief, meninges were removed in cold ACSF buffer (120 mM NaCl, 3 mM KCl, 2 mM MgCl, 0.2 mM CaCl, 26.2 mM NaHCO3, 11.1 mM glucose, 5 mM HEPES, 3 mM AP5, and 3 mM CNQX) bubbled with 95% oxygen. The tissue was dissociated with the papain dissociation kit (Worthington). Microglia were isolated using anti-CD11b + microbeads (Miltenyi Biotec).

### Histological analysis

Animals were perfused with 4% paraformaldehyde solution and cryopreserved. Tissues were paraffin-embedded and 20 µm sections were stained with hematoxylin and eosin at the Cancer Mouse Models Core Facility (VCU). For Luxol fast blue staining, paraffin embedded lumbar spinal cord sections were deparaffinized in xylene and stained with Luxol fast blue overnight at 60^0^C. The sections were differentiated in lithium carbonate and 70% ethanol until the gray matter was clear and white matter was sharply defined. Sections were counterstained with hematoxylin. The images were taken on Zeiss Axio imager A1 microscope and processed using Zen 2012 Blue acquisition software (Zeiss Inc.). Quantitative analysis was carried out using Image J software.

### Immunofluorescence

Animals were perfused with 4% paraformaldehyde, cryopreserved in 30% sucrose for 48 h, the lumbar region of the spinal cords were embedded in Tissue-Tek OCT Compound (VWR), and 30 µm frozen sections of L2–L4 spinal cords were prepared. For antibody staining, sections were incubated with primary anti-GFAP (Cell Signaling Technology; 1:300), anti-IBA (Wako Chemicals; 1:1000), anti-CC1 (Millipore; 1:300), anti-caspase-8 (Santa Cruz Biotechnology; 1:1000), or anti-NLRP3 (AdipoGen; 1:300) antibodies overnight at 4 °C. Subsequently, sections were incubated with Alexa Fluor 488 or Alexa Fluor 594 secondary antibodies (1:500, Invitrogen) for 1 h at room temperature. Nuclei were counterstained with Hoechst. Slides were mounted and sections were examined using Zeiss LSM 700 confocal microscope. Maximum projection images from confocal z-stacks were acquired. Care was taken to minimize pixel saturation while imaging each z-stack. Captured images were processed using Zen 2012 Blue acquisition software (Zeiss Inc.). Quantitative analysis of the cells was determined using Image J software. No fluorescence crossover was found between the channel and images were collected separately using appropriate laser excitation. Three–four animals per each group were examined and representative images are shown.

### Quantification of caspase-8 activity in spinal cord tissue

Lumbar spinal cord tissue of naïve or day 12 of EAE WT and cIAP2^−/−^ mice were flash-frozen in liquid nitrogen, mechanically powdered, and resuspended in 450 μl of cold extraction buffer (20 mM HEPES, 150 mM NaCl, 1% Triton X-100, 0.1% SDS, and 1 mM EDTA pH 7.5). Samples were sonicated, centrifuged, and the supernatants (100 μg protein/assay) were used for the assessment of caspase-8 activity using Caspase-Glo^®^ 8 Assay kit (Promega). Results were expressed as relative luminescent units per 100 μg protein.

### Quantitative PCR

mRNA expression was analyzed as previously described [45]. Total RNA was prepared from flash-frozen lumbar spinal cords of naïve (Figs. 1C, D and 5B) or day 12 of EAE (Figs. 1C, D and 4E, F) WT and cIAP2^−/−^ mice were flash-frozen tissue using Trizol (Life Technologies), reverse transcribed with the high-capacity cDNA kit (Applied Biosystems), and amplified on the BioRad CFXConnect Real-time System. SYBR Green intron-spanning pre-design qPCR primers (BioRad) were used. Gene expression levels were normalized to GAPDH and represented as fold expression over control.

### Isolation and culturing of primary cells from mice brains

Primary mixed glial cultures were prepared from WT and cIAP2^−/−^ P0–P3 pups. Briefly, brains were aseptically dissected, and meninges were removed. The tissue was mechanically dissociated, incubated with trypsin and DNaseI at 37 °C for 30 min, and centrifuged. Suspensions were filtered through 70 µM nylon cell strainers to obtain single-cell suspensions. Cells were resuspended and plated in dishes pre-coated with poly-d-lysine in Dulbecco’s modified Eagle's medium supplemented with 10% fetal bovine serum, penicillin/streptomycin, sodium pyruvate, and non-essential amino acids. The culture was fed after every 2 days for 8–9 days. The flask was shaken at 120 rpm for 2 h to isolate microglia.

### TUNEL assay

Lumber tissue was processed for terminal deoxynucleotidyl transferase biotin–dUTP nickend labeling (TUNEL) as per as manufacture’s protocol (Roche), and co-stained with either anti-Iba1 or anti-CC1 antibodies. Images of lumbar spinal cord tissue of day 12 of EAE WT and cIAP2^−/−^ mice were either captured on Zeiss Axio imager A1 microscope or LSM 700 confocal microscope.

### Isolation of bone-marrow-derived macrophages (BMDM)

Bone-marrow cells were isolated from the femurs of WT and cIAP2^−/−^ mice and cultured in RPMI 1640 media containing 10% FBS. For differentiation, the media was supplemented with 30 ng/ml of M-CSF (Biolegend) for 5 days.

### Antigen presentation assay

BMDM were stimulated with 50 ng/ml IFN-γ for 24 h and then treated with 50 ng/ml OVA antigen conjugated with fluorochrome AF-647 for 24 h. Antigen presenting cells and MHC II expression were quantified (Biolegend) by flow cytometry.

### Lactate dehydrogenase assay

The mixed glial cells or microglia were treated with LPS (100 ng/ml), TNF-α (30 ng/ml), or IL-1β (20 ng/ml) for 24 h. The lactate released by the dead cells was quantified using LDH assay kit (Dojindo Molecular Technologies, Inc) as per the manufacturer’s protocol.

### Blood–brain barrier permeability assay

100 μl of sodium fluorescein dye (100 mg/ml) was administered intraperitoneally into WT and cIAP2^−/−^ mice (EAE, day 12). After 45 min, blood was collected by cardiac puncture. Mice were perfused with PBS and CNS tissues were harvested, homogenized in PBS, clarified by centrifugation, precipitated in 1% trichloroacetic acid, and neutralized with borate buffer. Fluorescence was excited at 485 nm and read at 528 nm using microplate reader (Parkin Elmer 2000). Fluorescein concentration was calculated from standard curve and tissue fluorescence values were normalized to plasma fluorescence value of the same mouse.

### Statistical analysis

Statistical analysis was performed using GraphPad Prism 7 software. Quantitative data are expressed as mean ± SEM or mean ± SD (as specified). Sample sizes are indicated in figure legends. Differences across groups with multiple comparisons were analyzed with Two-way ANOVA with Tukey’s Multiple Comparison with the significance level of *p* < 0.05 was considered statistically significant.

## Results

### Deletion of cIAP2 exacerbates the severity of EAE

Although cIAP1 and cIAP2 limit cell death and inflammation [35], they are also critical for the induction of proinflammatory responses, including activation of NF-κB and MAPK [14]. cIAP2 also controls activation of IRF1, IRF1-driven expression of chemokines, and recruitment of T cells into sites of inflammation [31]. Interestingly IRF1^−/−^ mice are resistant to experimental autoimmune encephalomyelitis (EAE) [46], a commonly used mouse model of MS [47]. Based on these previous reports, we expected that cIAP2^−/−^ mice may be protected from neuroinflammation associated with EAE. Although disease incidence was similar in WT and cIAP2^−/−^ mice (75–85%), cIAP2^−/−^ mice displayed more exacerbated EAE symptoms than the WT mice (Fig. 1A) with an earlier onset of the disease and attained the peak symptoms much faster (Fig. 1B). In addition, 40% of cIAP2^−/−^ mice died due to the progress of the disease, while WT mice survived for a prolonged time (Fig. 1B). Since expression of proinflammatory cytokines and chemokines is elevated in MS patients and can cause oligodendrocyte cell death and axonal damage [48–50], we examined cytokine mRNA levels in the spinal cords during EAE. In agreement with the higher clinical scores, levels of proinflammatory cytokine (Fig. 1C) and chemokine (Fig. 1D) mRNAs were significantly higher in cIAP2^−/−^ mice than in WT littermates. Concordant with higher clinical scores and elevated cytokine/chemokine expression, the cIAP2^−/−^ mice also displayed more robust infiltration of immune cells into the lumbar spinal cords during EAE than the WT littermates (Fig. 1E). While this difference was evident during EAE, no infiltration of immune cells was found in either naïve cIAP2^−/−^ or WT mice (Additional file 1: Fig. S1A). Subsequent flow cytometry analysis indicated increased numbers of brain-infiltrating CD4^+^ T cells during EAE in cIAP2^−/−^ mice (Fig. 1F). Furthermore, cIAP2^−/−^ mice exhibited more extensive demyelination during EAE than the WT littermates (Fig. 1G, H). Exacerbated inflammation and demyelination in cIAP2^−/−^ mice during EAE was accompanied by activation of astrocytes indicated by an increased glial fibrillary acidic protein (GFAP) staining in the cIAP2^−/−^ spinal cords (Fig. 1I). Importantly, naïve WT and cIAP2^−/−^ littermates displayed comparable spinal cord myelination (Additional file 1: Fig. S1B), activation of astrocytes (Additional file 1: Fig. S1C), and blood–brain barrier integrity (Additional file 1: Fig. S1D) indicating lack of preexisting differences. Altogether, deletion of cIAP2^−/−^ enhances EAE severity, increases CD4^+^ T cell infiltration, enhances proinflammatory cytokine expression, increases activation of astrocytes, and augments demyelination.

### Deletion of cIAP2 from non-hematopoietic cells promotes severity of EAE

**Fig. 2:**
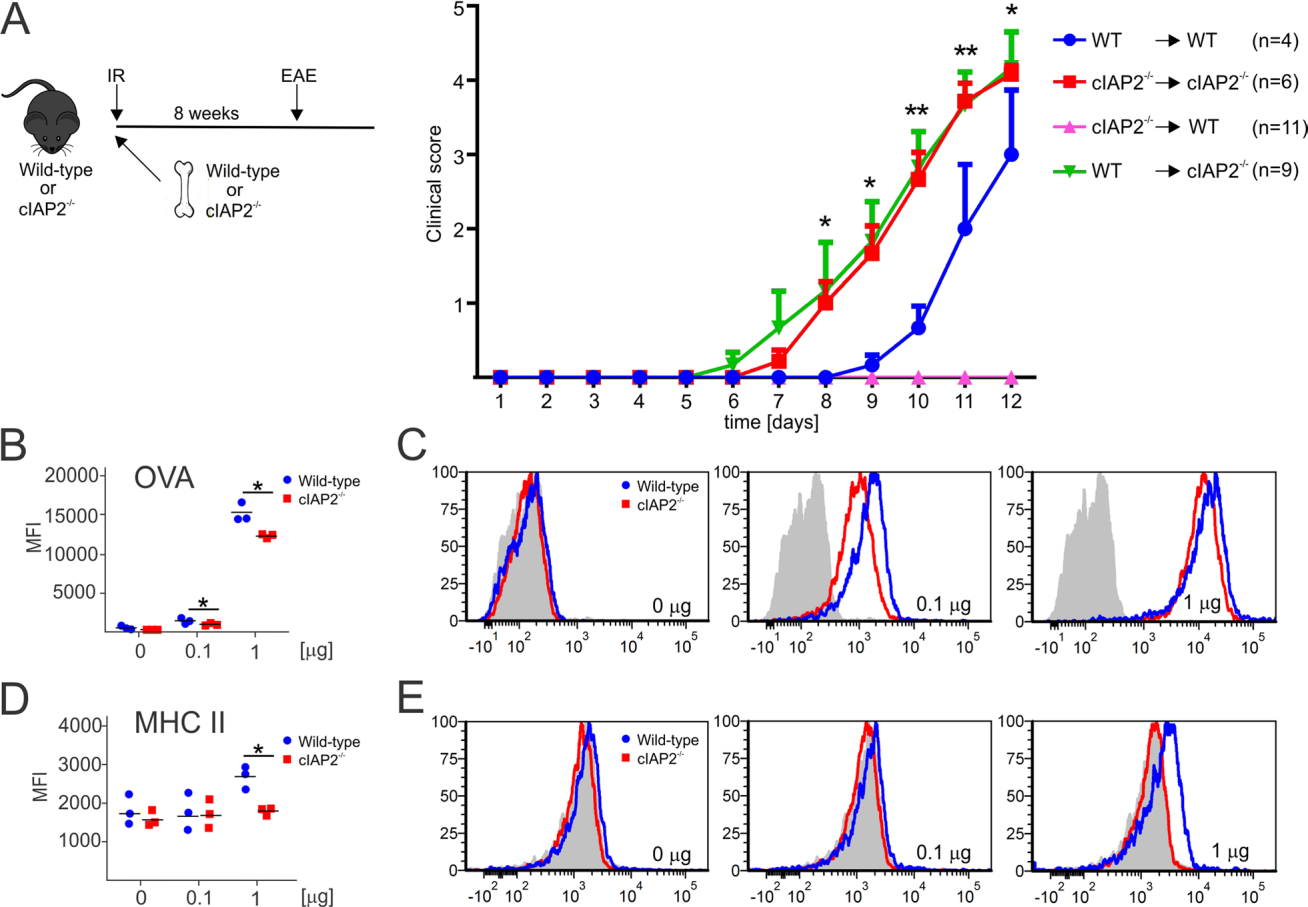
Exacerbated EAE disease is not caused by cIAP2^−/−^ bone-marrow derived cells. **A** Clinical score for EAE in WT → WT, (*n* = 4), cIAP2^−/−^ → WT (*n* = 11), WT → cIAP2^−/−^ (*n* = 9) and cIAP2^−/−^ → cIAP2^−/−^ (*n* = 6) bone-marrow chimera; mean ± SEM, *p < 0.05, **p < 0.01; Two-way ANOVA. **B**, **C** Antigen uptake and **D**, **E** MHC II expression in WT (*n* = 3) and cIAP2^−/−^ (*N* = 3) bone-marrow-derived macrophages treated with IFNγ and AF647 OVA antigen; mean ± SD, *T*-test; *p < 0.05. Grey peaks represent wildtype macrophages without IFNγ treatment

Since deletion of cIAP2 increased severity of EAE, we sought to determine which cell types contribute to this phenotype. We turn our attention to bone-marrow-derived cells, because cIAPs negatively regulate NIK and restrict B and T cell activation [51–53]. We generated bone-marrow chimeras in which only the hematopoietic or non-hematopoietic compartment expressed cIAP2 (Additional file 2: Fig. S2A). The cIAP2^−/−^ chimeras receiving either WT (WT- > cIAP2^−/−^) or cIAP2^−/−^ (cIAP2^−/−^- > cIAP2^−/−^) bone marrow rapidly displayed symptoms of EAE that were more severe than in WT chimeras receiving WT bone marrow (WT- > WT) (Fig. 2A). This suggests that cIAP2 in non-hematopoietic cells is primarily required to protect mice during EAE. Surprisingly, cIAP2^−/−^ bone marrow delayed the onset of the disease in WT chimeras (cIAP2^−/−^- > WT) (Fig. 2A) but the severity of the disease was comparable to that in WT chimeras receiving WT bone marrow (WT- > WT) at the later timepoints (Additional file 2: Fig. S2B). The delayed onset of the disease in the cIAP2^−/−^- > WT chimeras suggested a partial dysfunction of cIAP2^−/−^-derived bone-marrow cells. Since proliferation and activation of T cells requires antigen presentation [54], we compared antigen presentation and MHCII expression by cIAP2^−/−^ and WT BMDM. The uptake of the antigen by cIAP2^−/−^ BMDM was significantly reduced (Fig. 2B, C), and these cells also expressed MHCII at lower levels (Fig. 2D, E) suggesting that cIAP2 is needed for optimal antigen presentation. We conclude that the severity of EAE in cIAP2^−/−^ mice is driven by cIAP2-deficient non-hematopoietic cells. However, in contrast to this finding, cIAP2 deficiency in macrophages impairs antigen presentation, which can delay disease onset.

### cIAP2 deficiency limits the survival of glial cells in vivo and in vitro

**Fig. 3:**
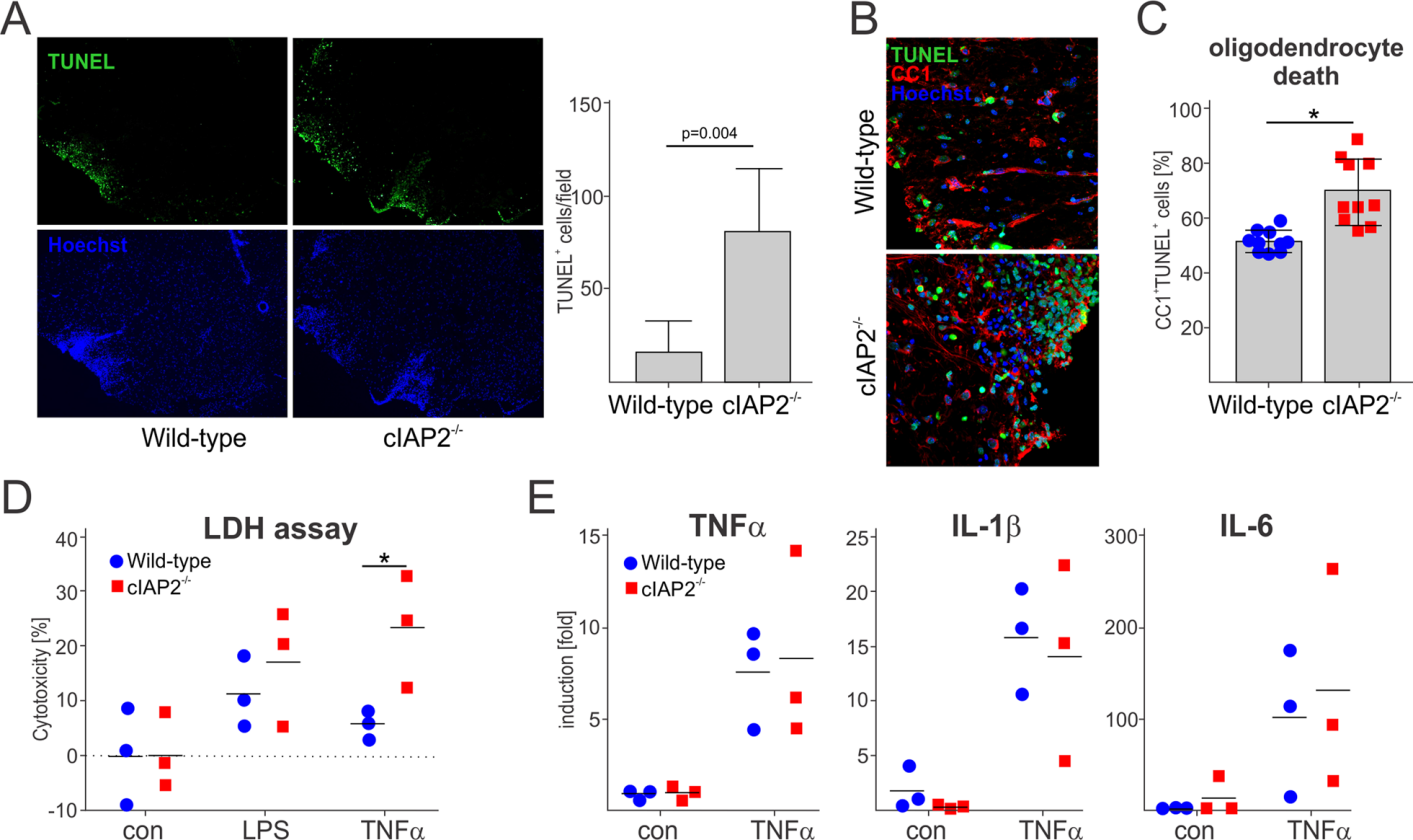
Increased death of oligodendrocytes in the spinal cords of cIAP2^−/−^ mice. TUNEL (**A**) and TUNEL (Quantification: *n* = 7, 7). and CC1 double staining. Quantification: (*n* = 10, 10). **B** of lumbar spinal cords (L2-L4) collected at day 12 of disease. The quantification of oligodendrocyte death is shown in **C**. **D** Cytotoxicity of mixed glial cultures treated with LPS and TNFα for 24 h (*n* = 3, 3). **E** Expression of proinflammatory cytokines by cultured microglia untreated and TNFα stimulated (4 h) (*n* = 3, 3). **C–E** Mean ± SEM, *p < 0.05, *T*-test

Expression of cIAP2 is upregulated during inflammation [36–38], which supports the pro-survival NF-kB signaling. Conversely, cIAP2 deficiency leads to cell death via caspase-8-dependent apoptosis or RIPK3-dependent necroptosis [9, 55, 56]. In MS, death of oligodendrocytes has been attributed to both apoptosis [57] and necroptosis [56]. As expected, we found significant amounts of TUNEL positive dying cells in the spinal cords of both WT and cIAP2^−/−^ mice during EAE (Fig. 3A). Most of these dying cells were oligodendrocytes, since they were also positive for the oligodendrocyte marker CCI (Fig. 3B). In agreement with exacerbated inflammation, demyelination, and disease severity, the numbers of TUNEL positive oligodendrocytes in cIAP2^−/−^ mice were significantly higher than in the WT littermates (Fig. 3C). Subsequently, we examined cell death of primary mixed glial cultures exposed to TNFα, which is abundantly expressed during EAE. LPS was also used because of the previously reported sensitivity of cIAP2^−/−^ macrophages [39]. Overall, cIAP2^−/−^ mixed glial cultures exhibited increased sensitivity to these stimuli, which was statistically significant for TNFα (Fig. 3D). We also examined cytokine expression in TNFα-stimulated microglia cultures; however, there was no difference in the expression of major proinflammatory cytokine mRNAs (Fig. 3E). These, data suggest that cIAP2 protects cells from cell death during EAE.

### cIAP2 restricts activation of microglia during EAE

**Fig. 4:**
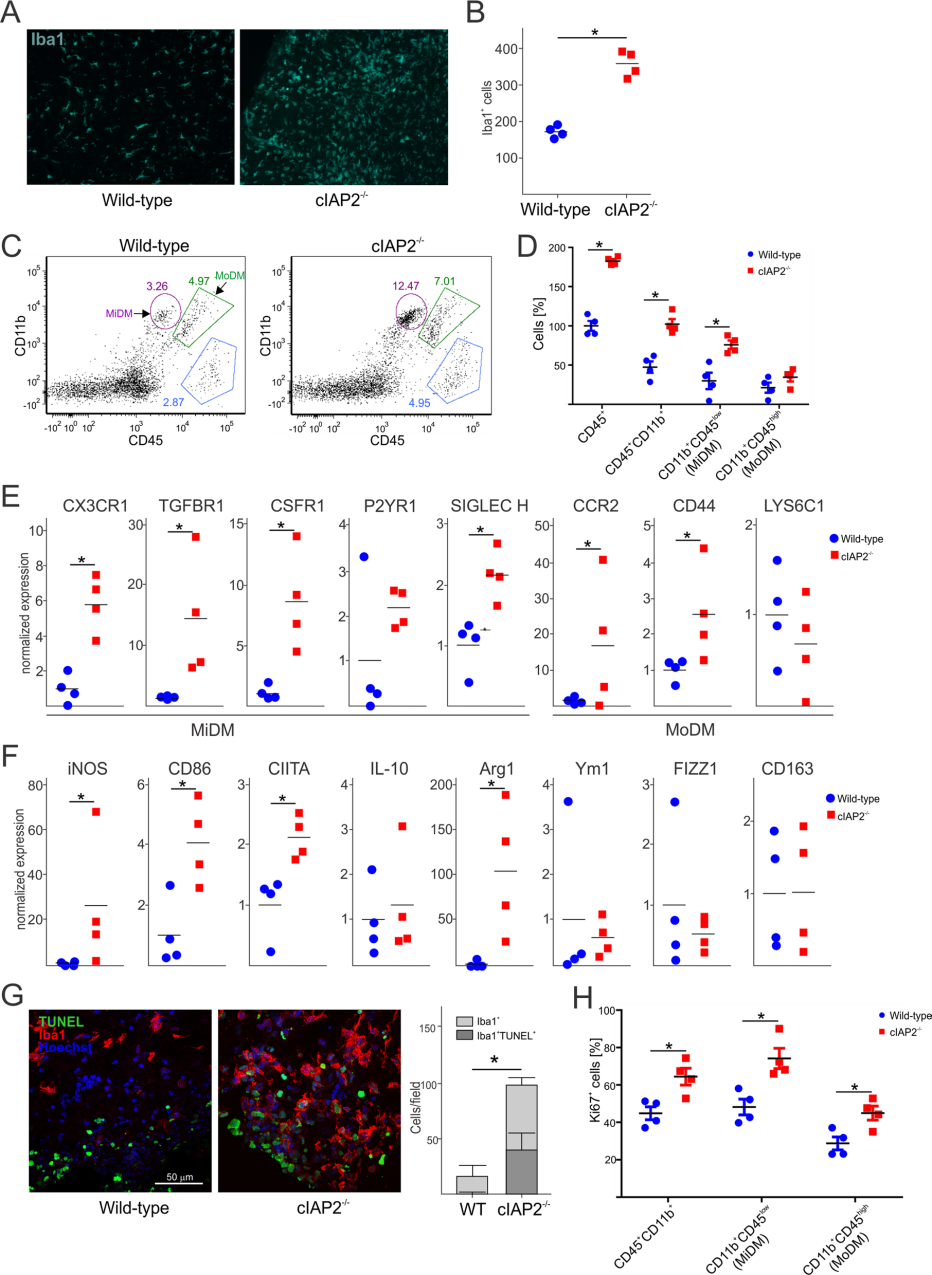
cIAP2 restricts activation of microglia during EAE. **A** Iba1 staining of lumbar spinal cords (L2-L4) collected at day 12 of disease. The quantification is shown in **B**. **C** Flow cytometry gating of myeloid cells in the WT (*n* = 4) and cIAP2^−/−^ (*n* = 4) brains of mice at day 12 of EAE disease. The quantification is shown in **D**. **E** Expression of markers of microglia-derived macrophages (MiDM), monocyte-derived macrophages (MoDM), and markers of polarization in the WT and cIAP2^−/−^ (*n* = 4, 4) lumbar spinal cords (L2-L4) collected at day 12 of disease. **G** TUNEL and Iba1 double staining of lumbar spinal cords (L2-L4) collected at day 12 of disease. (Quantification; *n* = 4, 4). **H** The quantification of flow cytometry of Ki67-positive cells in the WT and cIAP2^−/−^ (*n* = 4, 4) brains at day 12 of EAE disease. **B–H** Mean ± SEM, *p < 0.05, *T*-test

Microglia and blood-derived macrophages are critical for the pathogenesis of both MS and EAE [58]. Although the number and morphology of myeloid cells in the spinal cords of naïve WT and cIAP2^−/−^ littermates were comparable (Additional file 3: Fig. S3A), the number of activated myeloid cells that lost their characteristic dendritic processes were significantly higher in cIAP2^−/−^ spinal cords during EAE (Fig. 4A, B). It is established that EAE-induced activated myeloid cells represent both CD45^low^/CD11b^+^ microglia-derived macrophages (MiDM) and CD45 ^high^/CD11b^+^ monocyte-derived macrophages (MoDM) [43, 59]. The numbers of myeloid cells were comparable in naïve WT and cIAP2^−/−^ littermates and these cells were mostly resting microglia (Additional file 3: Fig. S3B), indicating that deletion of cIAP2 does not exhibit effects in naïve animals. However, the numbers of MiDM were nearly three times higher in cIAP2^−/−^ mice than in the WT littermates during EAE with the numbers of MoDM also higher but not statistically significant (Fig. 4C, D). Since MoDM and MiDM have different gene expression profiles [60], we analyzed expression of their specific markers during EAE. In agreement with drastically increased numbers of MiDM, expression of MiDM-specific mRNAs was significantly increased in the of cIAP2^−/−^ spinal cords (Fig. 4E). Although the numbers of MoDM were only moderately increased, some of the markers of MoDM were expressed at higher levels (Fig. 4E). To define whether cIAP2 deletion affects the polarization of activated myeloid cells, we examined expression of pro-inflammatory (M1) and anti-inflammatory (M2) markers in the spinal cords during EAE. In addition to abundantly expressed proinflammatory cytokines (Fig. 1C), we found increased expression of iNOS, CIITA, and CD86 (Fig. 4F) indicating a proinflammatory M1 phenotype. Interestingly, although expression of M2 specific markers, such as IL-10 and CD163, were not affected in cIAP2^−/−^ spinal cords, arginase expression was drastically increased (Fig. 4F). This corroborated previous reports that arginase expression is enhanced during acute EAE [61]. Subsequently, we examined whether cIAP2-deficient myeloid cells in the CNS are more prone to cell death during EAE, since cIAP2-deficient macrophages are more susceptible to cell death [39]. Although we found increased number of TUNEL-positive myeloid cells in cIAP2^−/−^ spinal cords during EAE, they had ameboid morphology indicating their activation rather than cell death (Fig. 4G), These cells seemed to be engulfing TUNEL-positive oligodendrocytes. Indeed, we found that MiDM as well as MoDM were proliferating as indicated by Ki67 staining (Fig. 4H). We conclude that cIAP2 limits proliferation and activation of brain myeloid cells, mostly microglia, during EAE.

### Increased caspase 8 expression and activation in cIAP2^−/−^ microglia during EAE

**Fig. 5:**
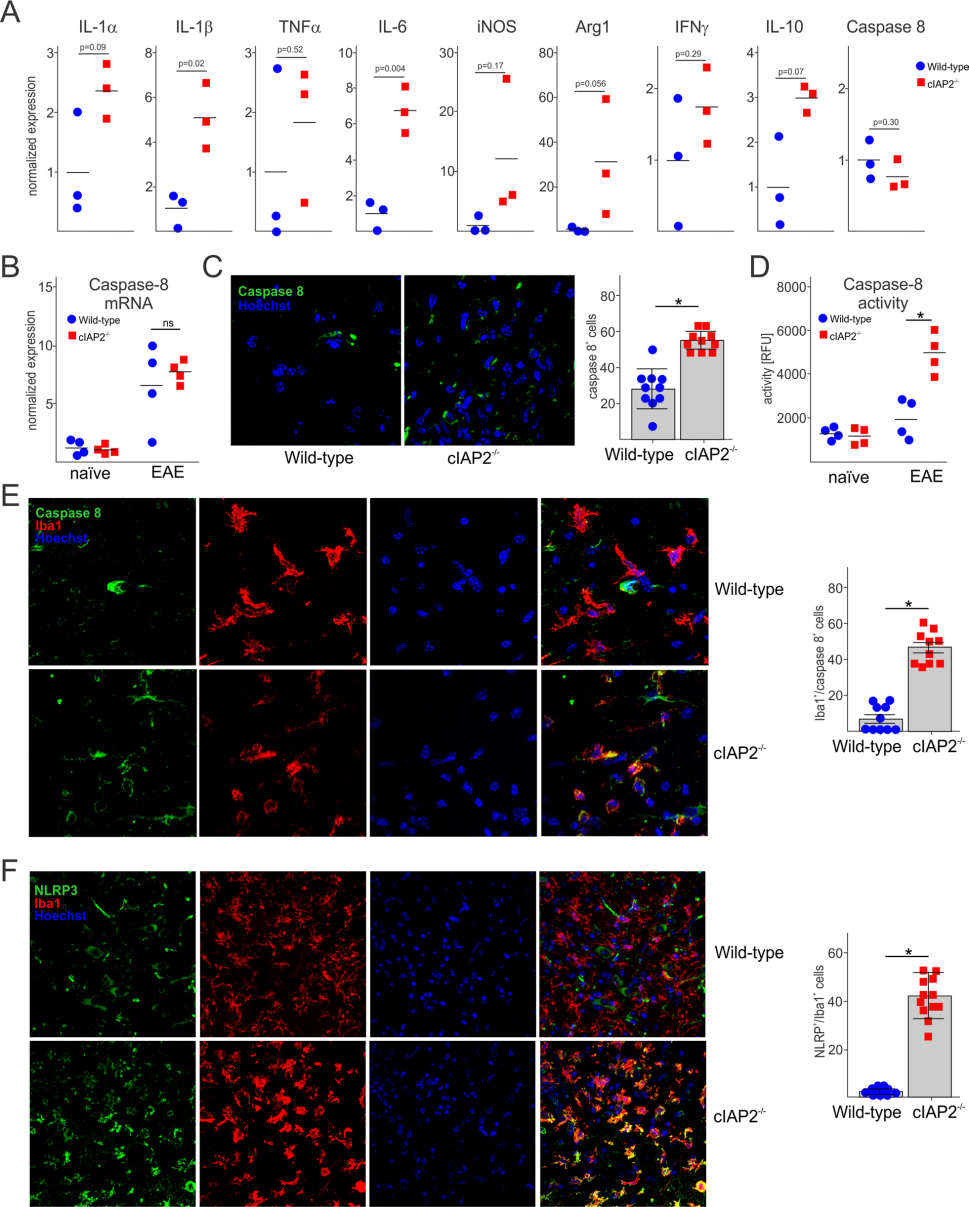
Increased caspase 8 expression and activation in cIAP2^−/−^ microglia during EAE. **A** Inflammatory gene expression in microglia isolated from WT and cIAP2^−/−^ (*n* = 3, 3) brains at day 12 of EAE disease. **B** Caspase-8 expression in naïve (*n* = 4, 4) and EAE (*n* = 4, 4) lumbar spinal cords (L2-L4, day 12 of EAE). **C** Caspase-8 staining of lumbar spinal (L2–L4) cords at day 12 of disease (left panels) and quantification (right panel). **D** Caspase-8 activity in lumbar spinal cords (L2–L4) at day 12 of disease. Caspase-8 and Iba1 **E** and NLRP3 and Iba1 **F** double staining of lumbar spinal cords (L2–L4) collected at day 12 of disease (left panels) and quantification (right panel). **A**–**F** Mean ± SEM, *p < 0.05, *T* test

Since expression of inflammatory mediators and markers is significantly upregulated in cIAP2^−/−^ spinal cords during EAE (Figs. 1C, D and 4E, F), we examined whether this increase can be attributed to the microglia. Indeed, expression of most of these markers, including IL-1α, IL-1β, TNFα, IL-6, iNOS, and Arg1, was significantly higher in cIAP2^−/−^ than WT microglia isolated from spinal cords of animals with EAE (Fig. 5A). We also analyzed expression of caspase-8, since it is a critical regulator of cell death and death-associated inflammation [35, 62]. However, caspase-8 mRNA levels were similar in WT or cIAP2^−/−^ microglia during EAE (Fig. 5A). In agreement, caspase-8 mRNA expression was also similarly upregulated in the WT and cIAP2^−/−^ spinal cords during EAE (Fig. 5B). In contrast to mRNA, expression of caspase-8 protein was significantly increased only in cIAP2^−/−^ spinal cords during EAE (Fig. 5C). The higher levels of caspase-8 protein in the cIAP2^−/−^ spinal cords correlated with increased caspase-8 activity (Fig. 5D). Caspase-8 has been reported to play an important role in the activation of microglia [63–66]. We explored whether cIAP2 deficiency leads to caspase-8 accumulation in myeloid cells during EAE. We found fivefold increase in the numbers of caspase-8-positive myeloid cells in the spinal cords of cIAP2^−/−^ mice during EAE (Fig. 5E). Importantly, caspase-8 is involved in controlling inflammation by regulating IL-1β expression and inflammasome activation (reviewed in [62]). More recently, caspase-8 activation by a non-canonical inflammasome [containing NLR family pyrin domain containing 3 (NLRP3), IRAKM, and ASC] has been shown to be critical for autocrine IL-1β-driven proliferation and activation of microglia as well as amplified cytokine production during EAE [66]. We found that the numbers of NLRP3 positive myeloid cells are much higher in the cIAP2^−/−^ spinal cords during EAE (Fig. 5F). These findings suggest that cIAP2 may inhibit non-canonical NLRP3/caspase-8-dependent myeloid cell activation during EAE thus restricting inflammation and the severity of the disease.

## Discussion

The proinflammatory role of cIAP2 in innate immune cells has been well-documented and involves multiple mechanisms. cIAP2 supports activation of the canonical NF-κB pathway, the MAPK pathway, and inflammasomes [14]. These are all critical to initiate responses of the immune cell after detection of pathogens. cIAP2 also limits apoptotic and necroptotic cell death of innate immune cells during their activation [14]. In contrast, the precise role of cIAP2 during adaptive immune responses is more elusive. cIAP2 regulates the non-canonical NF-κB pathway, which regulates many processes, including generation and maintenance of effector and memory T cells [67] as well as the survival, maturation, and homeostasis of B cells in peripheral lymphoid organs [67]. Nevertheless, the role of cIAP2 in complex immune diseases, such as MS, that involve coordinated responses of innate, adaptive immune, and non-immune cells is enigmatic. Our studies suggest a critical role of cIAP2 in regulating neuroinflammation during EAE. Indeed, the data we collected suggest that cIAP2 is required to limit disease severity and associated neuroinflammation. Surprisingly, however, the deleterious effects of cIAP2 deletion seem not to involve cells that originate in the bone marrow, which include T and B cells, as well as monocytes, since severity of the EAE was comparable in WT and cIAP2^−/−^ bone-marrow chimeras. Interestingly, cIAP2 deletion impaired activation of BMDM and their ability to express MHCII that is needed for antigen presentation. Decreased activation of BMDM is not surprising, since different aspects of antigen presentation are coordinately regulated by NF-κB [68–70]. Nevertheless, the decreased activation of cIAP2^−/−^ BMDM only resulted in the delayed onset of EAE. Furthermore, our studies indicate that cIAP2 limits EAE-associated neuroinflammation and disease severity by its specific actions in non-hematopoietic cells. Although our experimental setup does not allow to decisively identify the cell type(s) responsible for the observed phenotype, collectively the data point to microglia as a primary cell type driving the exacerbated neuroinflammation. We found that these cells become hyperactivated in cIAP2^−/−^ animals, which is associated with their increased proliferation, polarization towards M1 phenotype, and enhanced expression of proinflammatory mediators known to be associated with MS. However, in contrast to cIAP2^−/−^ macrophages [39] and cIAP2^−/−^ mixed glial cells in vitro (this study), cIAP2^−/−^ microglia seem to be resistant to neuroinflammation-associated cell death. Interestingly, activation of these cells coincided with elevated expression of caspase-8 and NLRP3.

Although caspase-8 plays a central role in initiating apoptosis and inhibiting necroptosis, it has well-documented functions that regulate inflammatory responses (reviewed in [62]). First, caspase-8 regulates IL-1β mRNA expression via activation of NF-κB [71–73]. Second, caspase-8 can directly cleave pro-IL-1β to active IL-1β in response to multiple stimuli. Third, caspase-8 can regulate both priming and activation of NLRP3 inflammasomes [74–76]. Fourth, caspase-8 can promote IL-1β secretion, which is RIPK3-dependent [33]; however, EAE severity is only minimally affected in RIPK3^−/−^ animals [77]. Fifth, caspase-8 can activate microglia, and its inhibition prevents microglia-mediated neurodegeneration [63, 65].

Recently, a non-canonical inflammasome, caspase-8/IRAKM/NLRP3/ASC complex, has been shown to assemble in microglia during EAE [66]. Processing of pro-IL-1β to active IL-1β by this non-canonical inflammasome and subsequent autocrine IL-1β stimulation has been proposed as a required step supporting microglia survival and proliferation during EAE. It remains to be established whether cIAP2 deficiency enhances activity of this non-canonical inflammasome and pro-IL-1β processing in microglia during EAE. Although cIAP2 and caspase-8 have not been shown to interact, TRAF2-mediated K48-linked degradative polyubiquitylation of caspase-8 has been reported [78]. As TRAF2 binds to cIAP1 and cIAP2 and serves as an adapter, it remains to be established whether caspase-8 is polyubiquitylated by the cIAP1/cIAP2/TRAF2 complex in activated microglia. While our data point to caspase-8-dependent microglia activation as a mechanism of increased neuroinflammation in cIAP2^−/−^ animals during EAE, it is possible that cIAP2 regulates other mechanisms in microglia. For example, the cIAP1/cIAP2/TRAF2 complex regulates the stability of c-Rel [30] and c-Rel is critical for the development of EAE with c-Rel^−/−^ mice protected from the disease [79]. RelB/p50 complexes are also known targets of the necroptosis-independent RIPK3-dependent pathway, promoting inflammation that could operate in microglia [80]. It is also possible that cIAP2 deficiency in other non-hematopoietic cells causes increased oligodendrocyte death and secondary neuroinflammation driven by overactivated microglia.

Since SMAC mimetics that target cIAPs are evaluated in multiple clinical trials for treatment of various cancers, they impact on neuroinflammation, and subsequent neurodegeneration needs to be carefully examined. Our data suggest that although these drugs are well tolerated, they may activate microglia and have deleterious neuroinflammatory effects in patients.

## Conclusions

Overall, our data demonstrate that cIAP2 modulates neuroinflammation, and cell death and survival during EAE. Significantly, cIAP2 seems to be crucial to limit activation of microglia during EAE thus limiting associated neuroinflammation.

## Supplementary Information

**Additional file 1: Figure S1. **Characterization of naïve WT and cIAP2^−/−^ mice. Hematoxylin and eosin (A), Luxol Fast blue (B) and GFAP immunofluorescence (C) staining of naïve WT and cIAP2^−/−^ lumbar spinal cords (L2–L4) (Quantification; n = 4, 4). (D) Quantification of fluorescein sodium salt accumulation in different CNS regions of naive WT (n = 3) and cIAP2^−/−^ (n = 3) mice (*p < 0.05, *T* test).

**Additional file 2: Figure S2. **EAE in bone-marrow chimeras. (A) PCR analysis of DNA isolated from either bone-marrow-derived cells or tails of mice. (B) Clinical score for EAE in WT → WT, (n = 6), cIAP2^−/−^ → WT (n = 6) bone-marrow chimera recorded for 17 days, mean ± SEM, *p < 0.05, **p < 0.01, ***p < 0.001, ****p < 0.0001, *T* test.

**Additional file 3: Figure S3. **Analysis of myeloid cells in naïve WT and cIAP2^−/−^ mice. (A) Iba1 staining of naïve WT and cIAP2^−/−^ lumbar spinal cords. (B) Quantification of immune cells in the brains of naïve WT (n = 4) and cIAP2^−/−^ (n = 4) mice, mean ± SEM, *p < 0.05, *T* test.

## Data Availability

Information regarding the experimental methods used, and the data in this paper are available to scientific communities upon direct contact to the authors. Individual requests for shipment of mice to AAALAC accredited institutions will be honored. An appropriately signed MTA will be required, as well as permission from original source of cIAP2^−/−^ mice (Dr. Korneluk).

## Abbreviations

BIR: Baculoviral IAP repeat
BMDM: Bone-marrow-derived macrophages
cIAP: Cellular inhibitor of apoptosis
CNS: Central nervous system
EAE: Experimental autoimmune encephalomyelitis
GFAP: Glial fibrillary acidic protein
IAPs: Inhibitors of apoptosis
IKKγ: Inhibitor of NF-κB kinase gamma
IRF1: Interferon regulatory factor 1
LPS: Lipopolysaccharide
MS: Multiple sclerosis
MAPK: Mitogen activated protein kinase
MiDM: CD45^low^/CD11b^+^ microglia-derived macrophages
MoDM: CD45^high^/CD11b^+^ monocyte-derived macrophages
NOD: Nucleotide-binding oligomerization domain
NF-κB: Nuclear factor kappa B
NIK: NF-kB-inducing kinase
NLRP3: NLR family pyrin domain containing 3
RIPK1: Receptor-interacting kinase 1
SMAC: Second mitochondrial-derived activator of caspases
TNFR: Tumor necrosis factor receptor 1
TLR: Toll-like receptors
TRAF2: TNFR-Associated Factor 2
TUNEL: Transferase biotin-dUTP nickend labeling
XIAP: X-linked IAP
