# Supplementary figures and images for: Cellular inhibitor of apoptosis 2 (cIAP2) restricts neuroinflammation during experimental autoimmune encephalomyelitis
Source: J Neuroinflammation. 2022 Jun 19;19:158. doi: 10.1186/s12974-022-02527-6 (PMC9208101; doi:10.1186/s12974-022-02527-6)

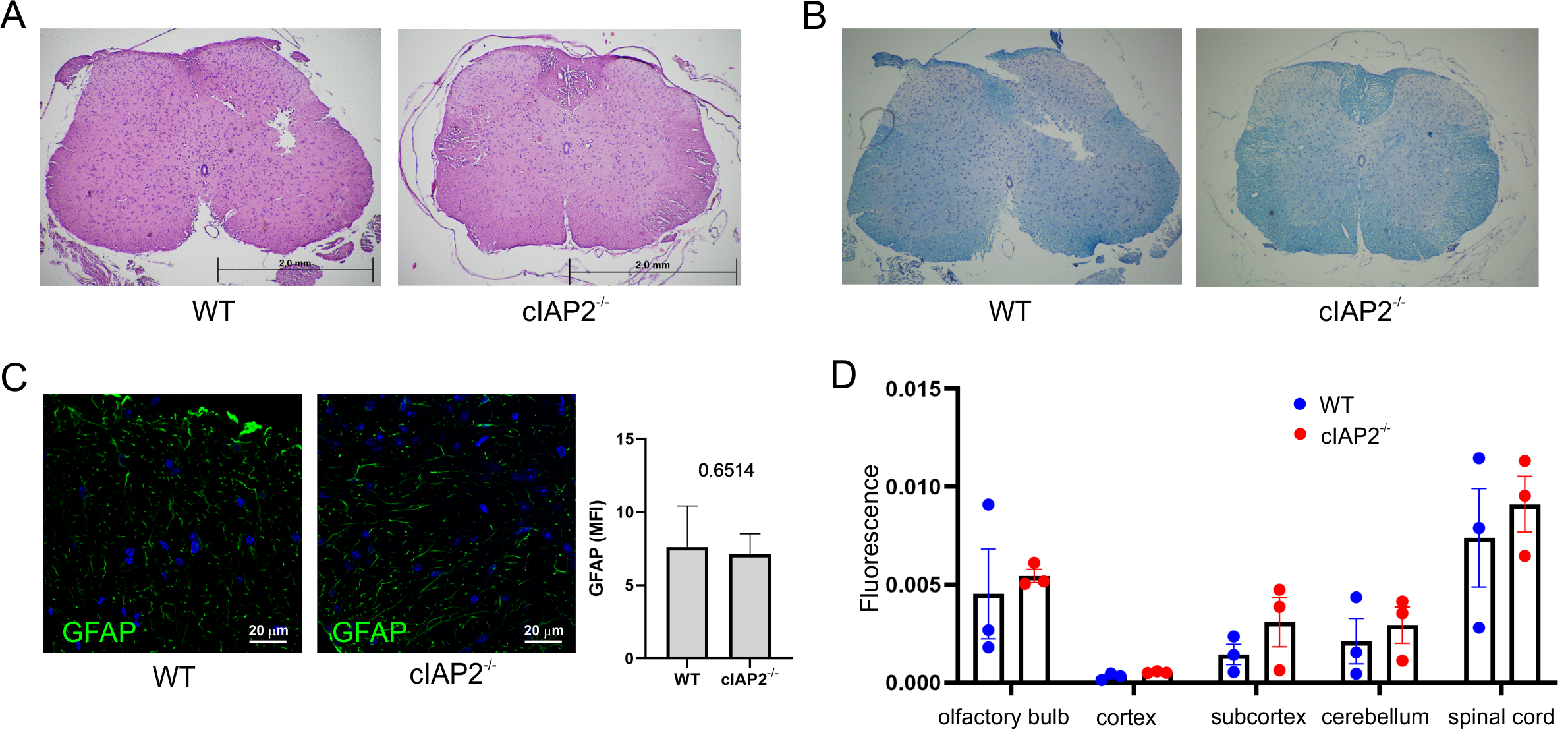

Supplement: Supplementary file 1 — Additional file 1: Figure S1. Characterization of naïve WT and cIAP2−/− mice. Hematoxylin and eosin (A), Luxol Fast blue (B) and GFAP immunofluorescence (C) staining of naïve WT and cIAP2−/− lumbar spinal cords (L2–L4) (Quantification; n = 4, 4). (D) Quantification of fluorescein sodium salt accumulation in different CNS regions of naive WT (n = 3) and cIAP2−/− (n = 3) mice (*p < 0.05, T test). [file 12974_2022_2527_MOESM1_ESM.tif]

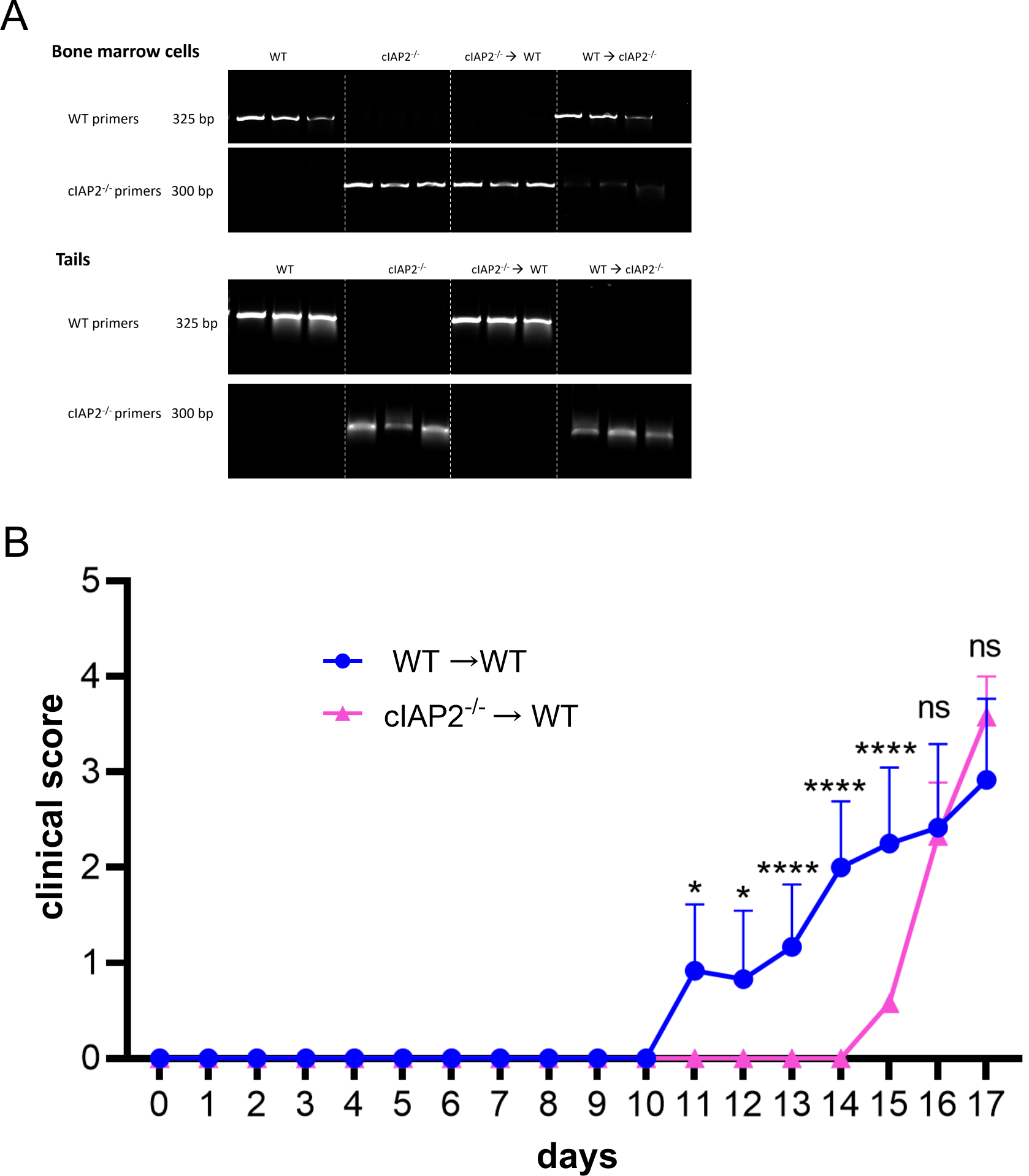

Supplement: Supplementary file 2 — Additional file 2: Figure S2. EAE in bone-marrow chimeras. (A) PCR analysis of DNA isolated from either bone-marrow-derived cells or tails of mice. (B) Clinical score for EAE in WT → WT, (n = 6), cIAP2−/− → WT (n = 6) bone-marrow chimera recorded for 17 days, mean ± SEM, *p < 0.05, **p < 0.01, ***p < 0.001, ****p < 0.0001, T test. [file 12974_2022_2527_MOESM2_ESM.tif]

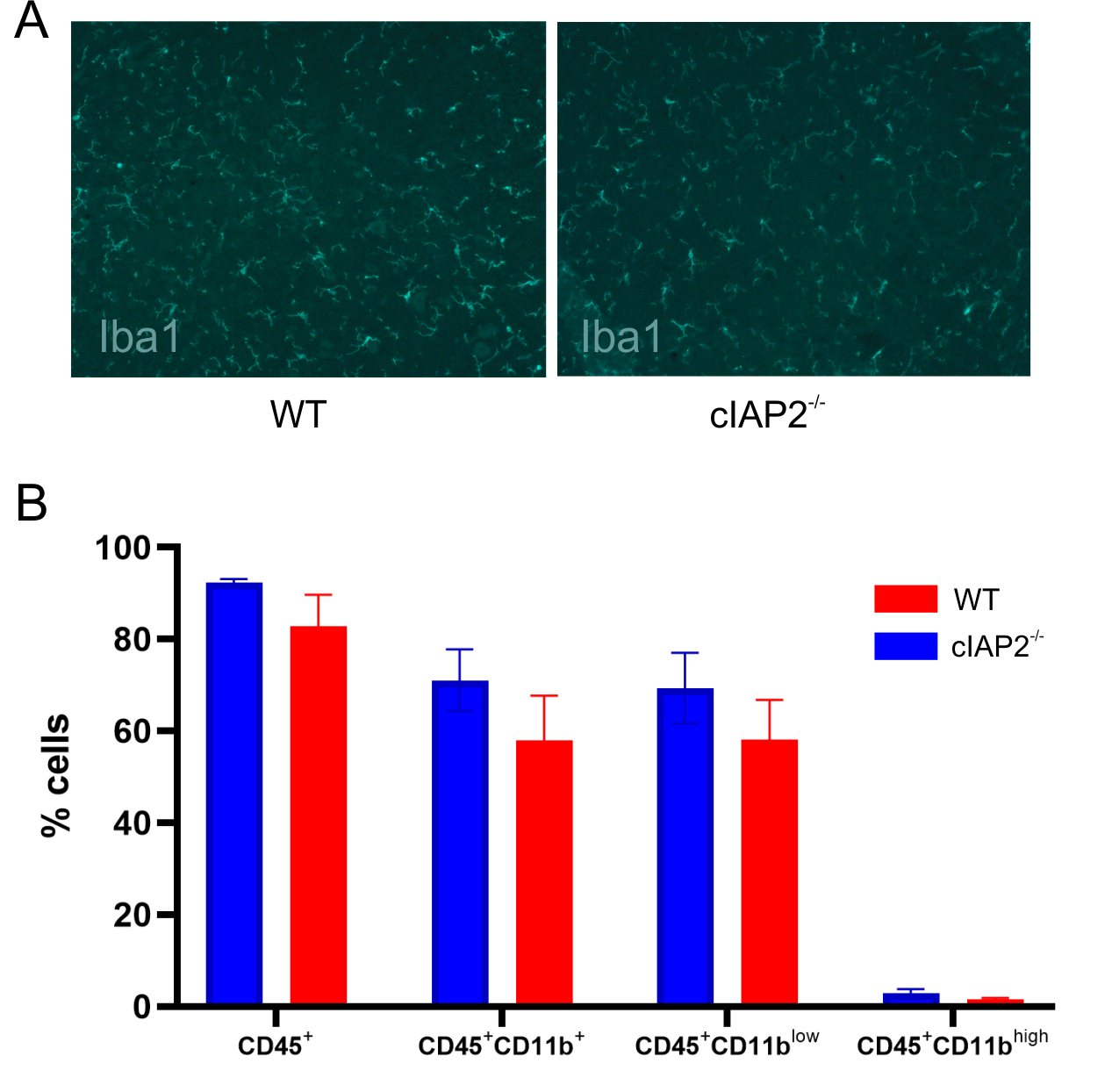

Supplement: Supplementary file 3 — Additional file 3: Figure S3. Analysis of myeloid cells in naïve WT and cIAP2−/− mice. (A) Iba1 staining of naïve WT and cIAP2−/− lumbar spinal cords. (B) Quantification of immune cells in the brains of naïve WT (n = 4) and cIAP2−/− (n = 4) mice, mean ± SEM, *p < 0.05, T test. [file 12974_2022_2527_MOESM3_ESM.tif]
